# Turnip mosaic virus infection cleaves MEDIATOR SUBUNIT16 in plants increasing plant susceptibility to the virus and its aphid vector *Myzus persicae*

**DOI:** 10.1186/s12870-025-06411-2

**Published:** 2025-04-02

**Authors:** Swayamjit Ray, Tyseen Murad, Gabriella D. Arena, Kanza Arshad, Zebulun Arendsee, Venura Herath, Steven A. Whitham, Clare L. Casteel

**Affiliations:** 1 Department of Plant Pathology and Plant-Microbe Biology, Cornell University, 309 Plant Science Building, Ithaca, NY 14853 USA; 2 Laboratório de Biologia Molecular Aplicada, Instituto Biológico de São Paulo, São Paulo, 04014-002 Brazil; 3 Interdepartmental Bioinformatics and Computational Biology Graduate Program, Iowa State University, Ames, IA USA; 4 Department of Agricultural Biology, Faculty of Agriculture, University of Peradeniya, Peradeniya, Sri Lanka; 5 Department of Plant Pathology, Entomology, and Microbiology, Iowa State University, Ames, IA USA

**Keywords:** Virus effector, NIa-pro, Aphids, Mediator 16, Plant defense

## Abstract

Plant viruses both trigger and inhibit host plant defense responses, including defenses that target their insect vectors, such as aphids. Turnip mosaic viru (TuMV) infection and its protein, NIa-Pro (nuclear inclusion protease a), suppress aphid-induced plant defenses, however the mechanisms of this suppression are still largely unknown. In this study, we determined that NIa-Pro’s protease activity is required to increase aphid performance on host plants and that 40 transcripts with predicted NIa-Pro cleavage sequences are regulated in Arabidopsis plants challenged with aphids and/or virus compared to healthy controls. One of the candidates, MEDIATOR 16 (MED16), regulates the transcription of ethylene (ET)/jasmonic acid (JA)-dependent defense responses against necrotrophic pathogens. We show that a nuclear localization signal is removed from MED16 by specific proteolytic cleavage in virus-infected plants and in plants overexpressing NIa-Pro in the presence of aphids. Although some cleavage was occasionally detected in the absence of virus infection, it occurred at a much higher rate in plants that were virus-infected or overexpressing NIa-Pro, especially when aphids were also present. This suggests MED16 functions in the nucleus may be impacted in virus infected plants. Consistent with this, induction of the MED16-dependent transcript of *PLANT DEFENSIN 1.2 (PDF1.2)*, was reduced in virus-infected plants and in plants expressing NIa-Pro compared to controls, but not in plants expressing NIa-Pro C151A that lacks its protease activity. Finally, we show the performance of both the virus and the aphid vector was enhanced on *med16* mutant Arabidopsis compared to controls. Overall, this study demonstrates MED16 regulates defense responses against both the virus and the aphid and provides insights into the mechanism by which TuMV suppresses anti-virus and anti-herbivore defenses.

**Supplementary Information:**

The online version contains supplementary material available at 10.1186/s12870-025-06411-2.

## Introduction

Proteins and small molecules that are used by pathogens and insect herbivores to successfully colonize a plant are known as effectors, and these molecules can also serve as reliable cues of attack that are recognized by the plant [1–3]. While plant recognition of effectors, effector-mediated suppression of plant defense, and plant counter-defenses have been extensively studied in recent years [2, 4, 5], the mechanistic details of multi-trophic and multi-player effector interactions are still not fully understood [3]. For example, plants are confronted with effectors from viruses and insect vectors during vector-borne virus transmission. In some cases, plant defense genes are activated by insect feeding exclusively, but trigger defenses that target both the viral pathogen and the insect vector, such as with the leucine-rich repeat receptor (NLR) gene *VAT-1* in melon [6]. There are also viral and insect effectors that can suppress plant defenses to increase the performance of a virus and the insect vector, such as with NIa-Pro (nuclear inclusion protease a) from turnip mosaic virus (TuMV) [7] and the whitefly salivary protein Bsp9 [8, 9]. On the other hand, some viral effectors antagonize vector performance while also benefiting the virus, such as 2b of cucumber mosaic virus (CMV) [10], further complicating the dialog of attack, recognition, and response.

The processes of protein metabolism and turnover play pivotal roles in plant defense and are often targeted by insects and pathogens [11–13]. For example, in response to TuMV infection, Arabidopsis plants downregulate plant protease gene expression while upregulating the expression of genes involved in autophagy and protein turnover (Bera et. al, 2022). Pathogens also counter plant defenses by interfering with protein turnover, often through interactions with the ubiquitin-proteasome complex [14–16]. Furthermore, when insects and pathogens induce jasmonic acid signaling, several negative regulators of the pathway are targeted for degradation by the ubiquitin-proteasome pathway [17]. This degradation process is thwarted by several viral effector proteins, such as C2 from tomato yellow leaf curl virus (TYLCV) and 2b from CMV, which stabilize the JAZ negative regulators, thereby blocking the downstream induction of plant defense [10, 18]. Viral effectors are also known to interact with the ubiquitin complex to mediate the suppression of host RNA silencing and target plant-defense related proteins for degradation [19].

Potyviruses encode a single polyprotein that must undergo precise cleavage events by viral-encoded proteases to coordinate different aspects of viral replication and proliferation in the host cell [20, 21]. Recently, it was shown that these viral proteases can also cleave plant proteins [22], a well-known phenomenon in animal-virus systems [23–25]. For instance, transient expression of an Arabidopsis histone reader protein (AtEML2), and a lysine ketoglutarate reductase (AtDUF707) in *Nicotiana benthamiana* are cleaved by NIa-Pro (nuclear inclusion protease a) from plum pox virus (PPV) and TuMV, both members of the *Potyviridae* family [22]. However, the specific function of these cleavages in plant-virus interactions was not determined. One example of NIa-Pro’s proteolytic activity directly affecting plant protein degradation was shown in *N.benthamiana,* where the host DEAD-box protein 5 (DBP5) was targeted by NIa-Pro for degradation leading to host cell death [26]. Additionally, it is estimated that NIa-Pro from TEV can interact with 76 different plant proteins [27], suggesting many unknown mechanisms still need to be identified.

TuMV infection and expression of its NIa-Pro suppress aphid-induced defenses downstream of the ethylene signaling in plants [7, 28, 29], but the mechanisms of this suppression are still largely unknown. In this study, we hypothesized that NIa-Pro may suppress plant defenses by cleaving plant proteins directly or in concert with another virus-induced plant protease that is required for aphid and virus defense induction. We used site-directed mutagenesis to abolish the protease activity of NIa-Pro, which prevented NIa-Pro from increasing aphid fecundity on host plants. To identify potential plant proteins that could be cleaved by NIa-Pro, we screened the transcriptome of virus-infected Arabidopsis plants with and without aphids. We found 40 candidate proteins, including MEDIATOR SUBUNIT16 (MED16). MED16 is known to regulate ethylene (ET)/jasmonic acid (JA)-dependent defense response in Arabidopsis (Wang et al., 2015). Using transient expression, stable transgenics, and immunoblots, we demonstrated that in the presence of NIa-Pro or TuMV and aphid feeding, MED16 abundance and its cleavage increases, while aphid induction of the MED16-dependent defense transcript *PLANT DEFENSIN 1.2* (*PDF1.2*) is suppressed [30]. Cleavage occurred largely in the cytoplasm and in the presence of virus and NIa-Pro but not in the presence of NIa-Pro C151A which lacks protease activity. This suggests that NIa-Pro mediated cleavage of MED16 protein disrupts MED16-dependent transcription by blocking the nuclear localization of MED16. In support of this, we found that both the virus and the aphid vector performed better on *med16* mutant Arabidopsis than control plants, indicating that MED16 plays a crucial role in the defense response against aphids and virus infection.

## Materials and methods

### Plants, insects, and virus infection

*Arabidopsis thaliana* (Col-0) and *Nicotiana benthamiana* seeds were obtained from Arabidopsis Biological Resource Center and Dr. Peter Moffett’s laboratory (Université de Sherbrooke, Quebec, Canada), respectively. Arabidopsis mutant line *med16* was obtained from Arabidopsis Biological Research Center (ABRC) and the seeds of MED16-FLAG complementation line in *med16* mutant background was generously provided by Dr. Zhonglin Mou at University of Florida [31]. Both Arabidopsis and *N.benthamiana* plants were grown in Cornell mix [by weight 56% peat moss, 35% vermiculite, 4% lime, 4% Osmocote slow-release fertilizer (Scotts, Marysville, OH, USA, http://www.scotts.com)] in 16:8 light:day cycle in growth chambers at 24°C. A tobacco (*Nicotiana tabacum*) adapted red strain of *Myzus persicae* colony was used for all experiments and was maintained on *Nicotiana tabacum* plants grown in the same growth conditions as stated above.

For virus infection, four-week-old Arabidopsis plants were rub-inoculated using stock plants of *N. benthamiana* infected with an infectious clone of TuMV (strain UK1 overexpressed inp35TuNOS) containing the green fluorescent protein sequence (TuMV-GFP) [32]. Inoculum was prepared by grinding one gram of fresh leaf tissue from *N. benthamiana* in 5 ml of 0.02M potassium phosphate buffer (pH = 5.2) on ice. The homogenized slurry was gently rubbed onto two rosette leaves of Arabidopsis plants with carborundum. As a control, inoculum was prepared from healthy *N. benthamiana* leaves and used to inoculate a second set of plants at the same time (mock-inoculated). *Agrobacterium tumefaciens* bacteria expressing TuMV-GFP was cultured overnight in Luria Bertani media, and cells were resuspended in 0.1M magnesium sulfate and 150µM acetyl syringone to obtain a final OD_600_ of 0.1. One milliliter of resuspended bacterial cells was then infiltrated into the ventral side of *N. benthamiana* leaves using a needle-less syringe. Plants that showed symptoms of viral infection and GFP expression after a week of infiltration were used as stock plants to rub-inoculate Arabidopsis plants.

### Cloning of constructs

The coding region of NIa-Pro protein was cloned from TuMV into the binary vector pMDC32 and the pSITE vector using Gibson assembly (New England Biolabs, USA) [33]. Protease activity of NIa-Pro was abolished by mutating cysteine 151 to alanine (C151A) using the same construct and the QuikChange Lightning Site-Directed Mutagenesis kit (Agilent, USA) in both pMDC32 and pSITE [22]. *A. tumefaciens* (GV3101 strain) was subsequently transformed with each construct. The viral RNA silencing suppressor protein P19 was cloned earlier in pMDC32 [13, 34].

### Expression of constructs in host plants

For stable expression, wild type Arabidopsis plants (Col-0 strain) were transformed with the empty plasmid vector pMDC32 (EV), pMDC32 NIa-Pro, or the pMDC32 NIa-Pro protease mutant C151A using the *A. tumefaciens* above and the floral dip method [35]. The seeds of the MED16-FLAG complemented lines were generously provided by Dr. Zhonglin Mou at the University of Florida, USA [31].

For transient expression of NIa-Pro, four-week-old *N. benthamiana* plants were infiltrated with overnight bacterial cultures of *A. tumefaciens* GV3101 cells carrying the pSITE empty vector, pSITE:NIa-Pro, or pSITE:NIa-Pro C151A mutant along with the pMDC32:P19 silencing suppressor. Bacteria were resuspended in 0.1M magnesium sulfate and 150µM acetyl syringone to obtain a final OD_600_ of 0.2. Plants were infiltrated with a needle-less syringe as described above.

To evaluate MED16 cleavage by NIa-Pro, the MED16-FLAG complemented plants were crossed with the Arabidopsis plants overexpressing the empty plasmid vector pMDC32, pMDC32 NIa-Pro, or the pMDC32 NIa-Pro (C151A). Pollen from the pMDC32 EV, NIa-Pro, or NIa-Pro C151 lines were used to pollinate MED16-FLAG plants. The F1 progeny were verified for the presence of NIa-Pro with RT-PCR, as described below in the RT-PCR section. F1 and subsequent F2 plants were self-crossed to obtain homozygous F3 progeny carrying MED-FLAG with pMDC32-EV, pMDC32-NIa-Pro, and pMDC32-NIa-Pro C151A mutant that were used to collect tissue for total protein extraction and nuclear separation.

### Aphid fecundity bioassays

For stable and transient expression experiments, one adult *M. persicae* was placed on a single leaf of each transgenic Arabidopsis plant or each agrobacterium-infiltrated *N. benthamiana* leaf 24 h after infiltration, using cages. Twenty-four hours later, all but one nymph was removed from the plant along with the adult aphid. The single nymph was allowed to develop on each plant or leaf for nine days for Arabidopsis and seven days for *N. benthamiana*, and then the number of aphids were counted. This was adequate time for the nymph to reach adulthood on each plant and to start producing their own nymphs. A similar setup was used for bioassays with wild type and the *med16* mutant plants (see methods below). For all fecundity experiments, at least 12 separate plants were used per treatment for each replicate, and each experiment was replicated at least twice.

### Screening the Arabidopsis transcriptome for NIa-Pro cleavage sites, MED16 nuclear localization signals (NLS), and MED16 isoforms

Recently, we characterized the transcriptome of Arabidopsis plants with and without TuMV infection and aphid infestation [13]. To identify putative host proteins potentially cleaved by NIa-Pro, protein sequences were obtained for all differentially expressed transcripts using the Arabidopsis proteome uniprot-proteome_UP000006548. Protein sequences were searched using the Perl *grep()* function with the search term corresponding to the most common NIa-Pro cleavage sequence (VxxQ). In contrast, the MED16 protein sequences were searched manually using the find function in Microsoft Word for the second most common cleavage sequence (VxxE) [36]. We used the NLStradamus tool from the University of Toronto to locate the nuclear localization signal of the MED16 protein sequences from Arabidopsis and *N. benthamiana.* We used Kallisto version 0.48.0 for the quantification of MED16 isoforms (Bray et al. 2016). First, a Kallisto index was constructed using the TAIR10 full-length cDNA obtained from Ensembl Plants (https://plants.ensembl.org/) release 107. Trimmed reads generated from each sample were pseudo-aligned to this index with 100 bootstraps using the following command: 'kallisto quant -b 100 –single -l 200 -s 20' to produce an abundance table with transcripts per million (TPM) values for each sample*.* We used I-TASSER for protein structure predictions of the different MED16 isoforms and solvent accessibility scores of the different NIa-Pro cleavage sites (Zhou et al., 2019; Zhou et al., 2022).

### MED16 transcript abundance and cleavage experiment set-up

Three-week-old Col-0 Arabidopsis plants were rub-inoculated with TuMV or mock-inoculated as described above. One week after rub inoculations, virus-infected plants were identified by using UV light to visualize the GFP. One cage was added to a single fully infected leaf for six TuMV-infected plants or to a single developmentally matched leaf from six mock-inoculated plants. Next, 20 adult aphids were added to each cage. As a control, cages were added without aphids to six additional plants for both treatments (virus- and mock-inoculated). The same methods were used to set up aphid and no aphid treatments on four-week-old Arabidopsis plants overexpressing pMDC32 EV, pMDC32:NIa-Pro, or pMDC32:NIa-Pro C151A protease mutant (*N* = 3–7 plants per treatment). Six to seven plants each were used for pMDC32 EV and pMDC32:NIa-Pro C151A with or without aphids, while four plants were used for pMDC32:NIa-Pro and three plants were used for pMDC32:NIa-Pro that were infested with aphids. For western blot analyses, plant tissue was collected from four-week-old Arabidopsis plants with or without aphid feeding that were overexpressing MED16-FLAG X pMDC32:EV, MED16-FLAG X pMDC32:NIa-Pro, or MED16-FLAG X pMDC32:NIa-Pro C151A mutant (pool of six plants per treatment; *N* = 6).

All cages and aphids were removed 48 h after infestation. Immediately after aphid and cage removal, tissue was collected, flash-frozen in liquid nitrogen, and stored at −80°C. For RNA extractions, 100mg of leaf tissue was collected separately from each plant replicate for each treatment. For total protein extractions, 50mg of tissue was collected from each plant for a total of 0.3g of pooled tissue per treatment. Similarly, another 0.3g of tissue was pooled for each of the treatments to perform nuclear protein extraction and separation.

### RNA extraction, cDNA synthesis, RT-PCR, and quantitative RT-PCR

Tissue was homogenized using liquid nitrogen and one metal bead of 1/8 inch diameter and shaken in a 5-gallon Harbil paint shaker (Fluid Management Inc, Wheeling, USA) for 20 secs three times while immersing tissue vials in liquid nitrogen between homogenization to prevent the tissue from thawing. Total RNA was extracted using the Quick- RNA kit (Zymo Research, CA, USA). One microgram of total RNA was used to synthesize cDNA using Oligo dT primers and Smart MMLV Reverse Transcriptase kit (Takara Bio Inc, Shiga, Japan). The expression of NIa-Pro was verified in all samples using gene-specific primers and RT-PCR (Fig S1a, Table S1). For quantitative RT-PCR (qRT-PCR), cDNA was diluted tenfold in nuclease-free water, and reactions were performed in a Bio-Rad CFX96 system using gene-specific primers (Table S1) and Bio-Rad SYBR green master mix (Bio-Rad, Hercules, USA). Each sample was run in triplicate. Cq values were exported and averaged across technical replicates for each sample, and then the relative expression of all treatments was calculated using the 2^–∆∆Ct^ method [37]. *UBIQUITIN* was used as the endogenous transcript control, after verifying the efficiency of the primer (Fig. S6), for the target genes *MED16, VSP2* and* PDF1.2,* and the undamaged plants that were mock-infested or undamaged plants of empty vector lines were used as the treatment controls*.*

The presence of NIa-Pro in virus-infected plants, pmDC32-NIa-Pro and pMDC32-NIa-Pro C151A overexpression plants, and MED16-3FLAG plants crossed with EV, pmDC32-NIa-Pro and pMDC32-NIa-Pro C151A overexpression plants was verified by the presence of NIa-Pro transcript using sequence specific primers sets (Table S1; Fig. S1). RNA was extracted from these plants and cDNA was made as described above which was then used as a template, and RT-PCR was performed using Go-Taq polymerase (Promega, WI, USA).

### Total protein extractions and immunoblot assays

Plant tissue was homogenized in a mortar and pestle with liquid nitrogen and total protein extracted in 3ml of ice-cold lysis buffer containing 0.5M sodium citrate, 5% sodium dodecyl sulfate, 1.5M sodium chloride, 2% ß-mercaptoethanol and 2 tablets/10ml of complete EDTA free protease inhibitor (Sigma Aldrich, USA), 100μM E-64 cysteine protease inhibitor [13]. Samples were then boiled at 100°C for 10min, followed by centrifugation at 14000g at room temperature for 10min in a tabletop centrifuge (Eppendorf 5425, Hamburg, Germany). The supernatant was collected and used to the quantify total extracted protein using a BCA protein quantification kit compatible with denaturing agents such as SDS and ß-mercaptoethanol (Thermo Fisher Scientific, Whatham, USA). Fifty micrograms of protein for each treatment were loaded onto a (4–20)% acrylamide SDS-PAGE denaturing protein gel (Bio-Rad, Hercules, USA) and separated at 4°C at 150 constant volts. Proteins were then transferred to a nitrocellulose membrane and blocked with 5% milk in 1 × Tris-buffered saline (TBS) for 2h. The blots were then incubated in antibodies overnight at 4°C in 5% milk in 1 × Tris-buffered saline. To visualize MED16-FLAG protein, a FLAG-HRP antibody from Mittenyl Biotech (Bergisch Gladbach, Germany) was used at 1:5000 dilution. Blots were visualized using the SuperSignal West Femto Maximum Sensitivity Substrate chemiluminescence kit (Thermo Scientific, Whatham, USA) on a Chemidoc (Biorad, Hercules, USA). Ponceau stains showing the 56kD subunit of Rubisco show equal protein loading in each well.

### Separation of nuclei-enriched and nuclei-free protein fractions and immunoblots

Tissue was homogenized in a mortar and pestle with liquid nitrogen, and 3ml of ice-cold lysis buffer was added consisting of 20mM Tris–HCl (pH = 7.4), 25% glycerol, 2mM EDTA, 25mM MgCl_2_, 40mM KCl, 250mM sucrose, 100μM E-64 and 1mM DTT [38]. The lysate was filtered through 100-micron and 40-micron filters sequentially on ice. The filtered lysate was centrifuged at 1500g for 10 min at 4°C, and the supernatant was stored as the nuclei-free fraction. The pellet consisting of the nuclei-enriched fraction was resuspended in nuclear resuspension buffer consisting of 20mM Tris–HCl (pH = 7.4), 25% glycerol, 25mM MgCl_2_, 100μM E-64 and 0.1% Tween-20 and centrifuged for 1500g for 10 min at 4°C to wash the nuclei-enriched fraction. The nuclei-enriched fraction was washed five times and then the nuclear pellet was weighed, and the pellet was resuspended in the nuclear resuspension buffer described above. The nuclei-enriched and nuclei-free fractions were boiled in 1X Laemmli buffer [39] for 10 min at 100°C, and protein was quantified using BCA compatible protein kit (Thermo Fisher Scientific, Whatham, USA). Fifty micrograms of protein were loaded onto a 4–20% gradient SDS-PAGE denaturing protein gel as described above, and immunoblot detection of MED16 protein was performed as described above using the anti-FLAG antibody. To evaluate successful nuclear and cytosolic protein separations, histone H3 and phosphoenol pyruvate carboxylase protein primary antibodies were used respectively in the ratio 1:10,000 (Agrisera biotech, Sweden). Histone proteins are localized only to the nucleus, while phosphenol pyruvate carboxylase is an enzyme that is involved in carbon metabolism in plants and is present only in the cytosol (Fig S2) [40, 41]. Anti-rabbit secondary antibody conjugated with horse radish peroxidase enzyme was used in the ratio of 1:10,000 (Sigma Aldrich, USA) and blots were visualized using chemiluminescent substrate as described above. The absence of Rubisco protein, which only localizes in the chloroplast stroma, in Ponceau stains in the nuclear fractions also shows that the nuclear fraction was not contaminated with chloroplasts. Band intensities were measured using the software ImageJ by selecting the area of the band in question and counting the number of gray pixels under that unit area. The intensities are reported in Table S2.

### Aphid fecundity and TuMV infection bioassays on med16 mutants

One adult *M. persicae* was placed on a single leaf of wildtype Col-0 and *med16* mutant Arabidopsis plants using 2cm diameter cages. Twenty-four hours later, all aphids were removed except for one nymph from each cage. Each remaining nymph was allowed to develop for nine days, and then the number of progeny and the founder counted. For TuMV infection bioassays, a single leaf of wildtype Col-0 and *med16* mutant Arabidopsis was inoculated with TuMV by rub inoculating Arabidopsis plants with leaves of *N.bethamiana* plants that were already infected with TuMV-GFP as described above. Ten days later, the number of plants with GFP visible in the rosette was counted as a measure of virus transmission in the plants. Ten separate plants of Col-0 and twelve plants of *med16* mutant were used for evaluating aphid fecundity, while 30 plants of each genotype were used to evaluate TuMV infection, and both experiments were replicated twice.

### Data analysis

Fecundity was analyzed by one-way ANOVA using the aov function in R (version RStudio 2022.07.2) at* p* value ≤ 0.05. Means separation was done using Tukey HSD post-hoc test using the TukeyHSD function in R. Two-way ANOVA tests were performed for gene expression data with the relative quantification (RQ) with insect presence and the plant treatment as the dependent variables. Multiple comparisons of means were done using post-hoc Tukey test using the emmeans function in R.

## Results

### The protease activity of NIa-Pro is required for increasing aphid performance on plants

**Fig. 1:**
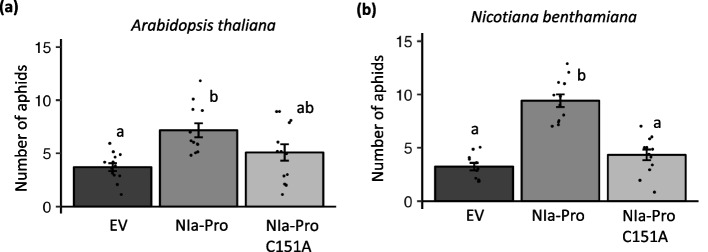
Protease activity of the viral protein NIa-Pro is required to increase aphid performance in plants. The number of progeny produced by a single one-day old *Myzus persica*e foundress after (**a**) 9 days on *Arabidopsis thaliana* or (**b**) 8 days on *Nicotiana benthamiana* plants overexpressing an empty expression plasmid (EV), NIa-Pro, or the NIa-Pro mutant (C151A) that lacks protease activity. Aphid reproduction was higher on plants expressing NIa-Pro compared to the EV control, while there was no difference in aphid progeny when feeding on NIa-Pro C151A compared to EV controls. Data were analyzed using one-way ANOVAs and means separation was performed using Tukey HSD test. Letters represent significant differences among treatments using a *p-*value < 0.05; error bars indicate standard error; *N* = 12

Transient expression of the viral protease NIa-Pro has been shown to inhibit plant defenses that target aphids and increase aphid performance [7]. To determine if the protease activity of NIa-Pro is required for this phenotype, we mutated the active site cysteine (C151) to alanine (C151A) to create a protease inactive version of the protein as previously described [22]. Consistent with our previous studies [7], stable expression of NIa-Pro in Arabidopsis (Fig. 1a; F_2,35_ = 7.74, *p* = 0.00165) and transient expression of NIa-Pro in *N. benthamiana* (Fig. 1b; F_2,32_ = 43.82, *p* < 0.0001) increased aphid fecundity (which is a marker of aphid performance) compared to plants expressing the empty expression vector. In contrast, expression of NIa-Pro C151A that lacks protease activity did not significantly enhance aphid fecundity compared to the controls in either host plant (Fig. 1a, b). This suggests that the protease activity of NIa-Pro is required for its ability to inhibit plant defenses and increase aphid performance*.*

### Aphid- and virus-induced Arabidopsis transcripts encode proteins with putative NIa-Pro cleavage sites

**Fig. 2:**
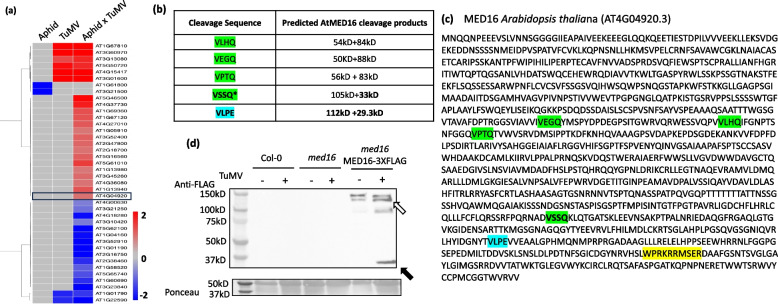
The MEDIATOR16 (MED16) protein is cleaved upstream of a nuclear localization signal (NLS) in the presence of turnip mosaic virus. **a** A heatmap showing differential expression of genes encoding proteins with predicted NIa-Pro cleavage sites (V-X–H-Q.) from plants infected with turnip mosaic virus (TuMV) and/or fed upon by *M. persicae* (aphid) compared to mock-inoculated, aphid-free plants. Med16 expression is highlighted in the black box. **b** The *Arabidopsis thaliana* MED16 protein sequence was searched for the two most common NIa-Pro cleavage sites (VxxQ & VxxE). Predicted cleavage product sizes are shown. **c** The location of the NIa-Pro cleavage sites (Green and Blue) and of the nuclear localization signal (NLS; Yellow) in the amino acid sequence are presented. **d** Col-0 wildtype Arabidopsis, *med16* mutants, and *med16* mutants complemented with MED16-3XFLAG were mock-inoculated or inoculated with turnip mosaic virus and then protein abundance of *MED16* was measured using immunoblots and FLAG antibodies. The full length MED16 protein (white arrows) was detected as well as a smaller ~ 36kDa putative cleaved product (black arrows). Each lane represents proteins extracted from 5 individual plants. Equal amounts of protein were loaded into each well as shown by Ponceau stain

Proteases from animal viruses can cleave host proteins with specific cleavage sequences to regulate host defenses [42, 43]. To investigate if the potyviral protease NIa-Pro could cleave plant proteins that are required for plant defenses, we searched for the most common NIa-Pro cleavage site (VxxQ and VxxE) in the protein sequences of the TuMV and/or *M. persicae* de-regulated Arabidopsis genes from a recent transcriptome study (Bera et al. 2023). Only 40 of the 1626 DEGs contained putative NIa-Pro cleavage sites in their predicted coding sequence across all treatments (Fig. 2a). The mRNA transcripts of most of the predicted plant proteins with putative NIa-Pro cleavage sites were differentially expressed in plants infected with TuMV and fed on by aphids (38 DEGs, TuMV x Aphid column; Fig. 2a). Roughly 50% of the 38 DEGs were up-regulated and down-regulated in response to TuMV infection and aphid feeding compared to controls (Fig. 2a; 16 down, 22 up, TuMV x Aphid column). Eight of the DEGs in plants infected with TuMV alone contained predicted NIa-Pro cleavage (Fig. 2a). All eight of these were also differentially regulated in plants infected with TuMV and fed upon by aphids (TuMV column & TuMV x Aphid; Fig. 2a), and they were regulated in the same direction and magnitude in both treatments, suggesting these are not impacted by aphid presence (Lawrence et al*.*, 2012 Fig. 2a; TuMV & TuMV x Aphid columns). Only two of the DEGs in response to aphid feeding alone encoded proteins with predicted NIa-Pro cleavage sites, and both were down-regulated compared to controls (Fig. 2a; Aphid column).

We searched the 40 candidates for genes already known to be involved in plant defense responses against aphids or virus infection. Among the genes, we found SYP122 (AT3G52400), a Qa-SNARE protein involved in callose deposition, vesicle fusion [44], and a known negative regulator of programmed cell death, salicylic acid signaling, and jasmonic acid signaling [45]. Recently, it was also shown that SYP122 transcripts are induced by *M. persicae* feeding on Arabidopsis plants [46]. TREHALOSE PHOSPHATASE SYNTHASE 11 (TPS11; AT2G18700), a protein shown previously to induce resistance to aphids in Arabidopsis [47], and RNASE-II like 1 (RTL1; AT4G15417), a protein that has been shown previously to suppress RNA silencing in host plants [48], were two other defense-related genes found among the candidates.

A particularly interesting defense-related candidate for us was SENSITIVE FOR FREEZING 6 (SFR6) or MEDIATOR 16 (ATG404920), a plant protein involved in regulating jasmonic acid and ethylene-dependent defense responses and resistance to necrotrophic pathogens (Fig, 2a, highlighted in black box) [31]. MED16 has also been shown to bind to the transcription factor WRKY33 [31, 49], an important negative regulator of ABA and SA responses and activator of ET-dependent plant defenses [50]. Previously, we demonstrated that aphid herbivory induces ethylene-dependent plant defenses that are inhibited in potyvirus-infected plants [28, 51]. This led us to hypothesize that potyvirus inhibition of ethylene-dependent defenses could be due to MED16 cleavage by NIa-Pro, and this could explain why aphid fecundity was lower in plants that expressed NIa-Pro that did not have protease activity (Fig. 1a, b).

### The MED16 protein has multiple putative NIa-Pro cleavage sites upstream of a nuclear localization signal

To determine if NIa-Pro cleaves MED16, we conducted immunoblots with *med16* mutant Arabidopsis that have been complemented with a FLAG-tagged version of MED16 with and without TuMV infection. In immunoblots, we detected two bands between 180 and 130 kD in all treatments, which corresponds to the size of MED16 and is consistent with previous studies (Fig. 2d; white arrow). We also detected a ~ 36kD protein product in the virus treatment (Fig. 2d; black arrow).

To investigate if this could be a putative cleavage product of NIa-Pro, we searched for the location of the Arabidopsis MED16 VxxQ and VxxE cleavage sequences [52]. We found three predicted isoforms of MED16 (Fig. S4), and each isoform sequence contained five potential VxxQ cleavage sites (Fig. 2b,c). We determined AT4G04920.3 was marginally more abundant than other isoforms of MED16 in control plants (Fig. S4a; *p* = 0.1) by reanalyzing an Arabidopsis transcriptome data set we recently published (Bera et al*.* 2021). To determine if all four predicted NIa-Pro cleavage sites were accessible, we used I-TASSER to predict the structure of the most abundant MED16 isoform (Fig. S4a), as well as the other two isoforms (Fig S4b). One site, VSSQ, was predicted to be more accessible than the other sites (Fig. 2b), and to produce a putative cleavage product around ~ 36kDA with the 3kDA FLAG tag, which corresponded to the immunoblot (Fig. 2d; black arrow). When comparing the predicted structures of the different isoforms, we noticed an open-hook like structure for AT4G04920.3, that was not present for the other two isoforms (Fig. 2d; Fig. S4b). Notably, aphid feeding reduced the abundance of this form of MED16 (Fig. S4a). However the functional relevance of this was not determined. To determine how cleavage could impact function, we next examined MED16 for functional motifs and localization signals and found a nuclear localization signal (NLS) present downstream of the potential NIa-Pro cleavage sites (Fig. 2c). These results suggest NIa-Pro may cleave off the NLS from MED16 along with the putative ~ 36kD cleavage product.

### Presence of aphids and virus increased MED16 transcript and protein levels and cytosolic cleavage of the MED16 protein

**Fig. 3:**
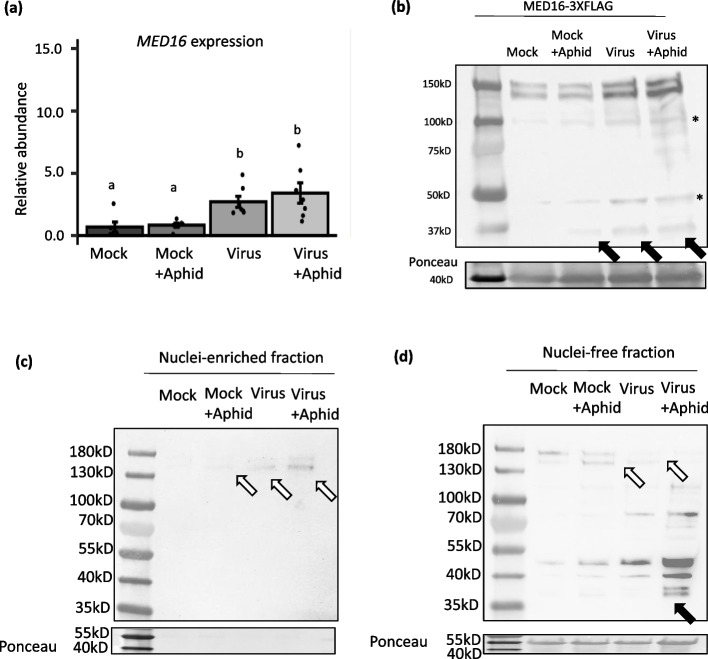
Aphid feeding in the presence of TuMV increased MED16 abundance and cleavage outside the nucleus. Relative expression of *MED16* transcript in (mock- and virus-infected plants with and without aphid feeding. *UBIQUITIN* was used as an endogenous control, while mock or EV treatments were used as the treatment controls for calculating relative expression (**a**). qRT-PCR data were analyzed using two-way ANOVAs and means separation was performed using Tukey test (*N* = 5 −7). Bars with different letters indicate significant differences at p-value < 0.05; error bars indicate standard error. Protein abundance of MED16 was measured in *med16* mutants that were complemented with MED16-3XFLAG using immunoblots and $\text{FLAG}$ antibodies in plants that were mock- and virus-infected with and without aphid feeding (**b**). Each lane represents proteins extracted from 5 individual plants. Equal amounts of protein were loaded into each well as shown by Ponceau stain. Non-specific bands are denoted by *. Immunoblot detection of the MED16 protein in the nuclei-enriched (**c**) and nuclear-free (**d**) fractions in *med16* mutant plants that were complemented with MED16-3XFLAG and were infected with turnip mosaic virus with and without aphid feeding. The ~ 36kDA putative cleaved product of MED16 is indicated by black arrows. Each lane represents proteins extracted from 6 individual plants. An equal amount of protein was loaded in each well as shown by Ponceau stain, and MED16 was detected using a FLAG antibody

We next evaluated the impact of aphid feeding and virus infection on *MED16* mRNA abundance in an independent experiment. Virus infection significantly increased *MED16* transcripts compared to controls (Fig. 3a; Main effect, virus: F_1,22_ = 18.007, *p*-value = 0.0003), however there were no significant impacts of aphid feeding or interactions between aphid and virus on *MED16* transcript accumulation (Fig. 3a; Main effect, aphid: F_1,22_ = 0.680, *p*-value = 0.418; virus x aphid: F_1,22_ = 0.262, *p*-value = 0.614). Upon performing means separation with a Tukey test between the treatments, we observed that *MED16* transcripts were higher in Arabidopsis plants at 10 days post TuMV infection (2.71 ± 0.444; Fig. 3a) and in virus infected-plants infested with aphids (3.41 ± 0.819; Fig. 3a), compared to mock-inoculated plants (0.686 ± 0.391; Fig. 3a) or mock-inoculated plants infested with aphids (0.833 ± 0.167; Fig. 3a). Notably, immunodetection showed MED16 protein abundance increased in virus-infected plants and in plants with aphids feeding compared to the mock controls (Fig. 3b). We also detected the ~ 36kDa product in the aphid and virus treatment individually and in the combined treatment (Fig. 3b; black arrows). Together, these results suggest MED16 is induced in the presence of aphids or TuMV, and protein cleavage is increased. As a transcription regulator, MED16’s primary functions occur in the nucleus. Aphid feeding and virus infection increased the accumulation of the full-length MED16 in the nucleus, while the combined treatments enhanced MED16 nuclear accumulation even more (Fig. 3c; white arrows, Table S2). Our previous results demonstrated that NIa-Pro is located inside the nucleus and cytosol, however it must be outside of the nucleus to inhibit plant defenses and enhance aphid fecundity [29]. This led us to hypothesize that MED16 may be cleaved in the cytosol in the presence of virus infection and aphid feeding before it is able to relocate to the nucleus. To address this hypothesis, we next measured the amount of full-length MED16 and MED16 cleavage products in the cytosol. Virus infection alone and in combination with aphid feeding reduced the accumulation of full-length MED16 in the cytosol (Fig. 3c,d; white arrows). While the full-length MED16 was barely present in the nuclei-free cytosolic fraction of plants with aphid and virus feeding, there was a significant increase in the putative MED16 cleaved products in the nuclei-free cytosolic fraction, including accumulation of the ~ 36 kD product (Fig. 3d; black arrows, Table S2). We also observe two cleaved MED16 protein bands that are ~ 36kD in the cytosolic fraction, which could be indicative of the MED16 isoforms that may be co-expressed in Arabidopsis (Fig S4) or due to modification of the cleaved product.

### TuMV infection suppresses MEDIATOR16-dependent transcript accumulation

**Fig. 4:**
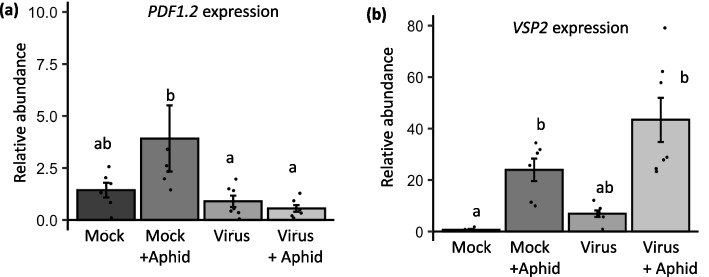
PDF1.2 is suppressed by virus infection, while VSP2 expression is only induced by aphids. Relative expression of *PDF1.2* and *VSP2* was measured in (**a**, **b**) mock and virus- infected plants with and without aphids. *UBIQUITIN* was used as an endogenous control. Mock treatments were used as the treatment controls for calculating relative expression. Two-way ANOVAs were conducted and means separation performed using Tukey HSD test for all experiments (*N* = 5—7 for a & b). Bars with different letters indicate significant differences at *p*-value < 0.05; error bars indicate standard error

If NIa-Pro MED16 cleavage reduces the pool of active MED16 in the nucleus, we hypothesized that MED16-dependent gene expression would also be reduced. To address this hypothesis, we measured the transcript abundance of *PLANT DEFENSIN 1.2* (*PDF1.2*), which has been shown previously to be MED16-dependent [31], and the abundance of a MED16-independent transcript, JA-dependent *VEGETATATIVE STORAGE PROTEIN2* or *VSP2* [53]. The expression of *PDF1.2 transcript* was suppressed overall in the presence of the virus when we used the presence or absence of the virus as the main effect in our two-way ANOVA model regardless of aphid feeding (main effect, virus: F_1,22_ = 6.493, *p*-value = 0.0183). On the other hand, aphid herbivory had no main effect on *PDF1.2* expression (main effect, aphid: F_1,22_ = 1.590, *p*-value = 0.2205). There was however, a marginally significant interacting effect of virus infection and aphid infestation (virus x aphid: F_1,22_ = 3.403, *p*-value = 0.0786). *PDF1.2* relative expression was highest in mock-inoculated plants with aphids feeding (3.92 ± 1.59). However, aphids were not able to induce *PDF1.2* expression in the virus-infected plants (0.594 ± 0.158; Fig. 4a). This suggests that pathogen-induced defenses in plants downstream of MED16 are suppressed in virus-infected plants.

**Fig. 5:**
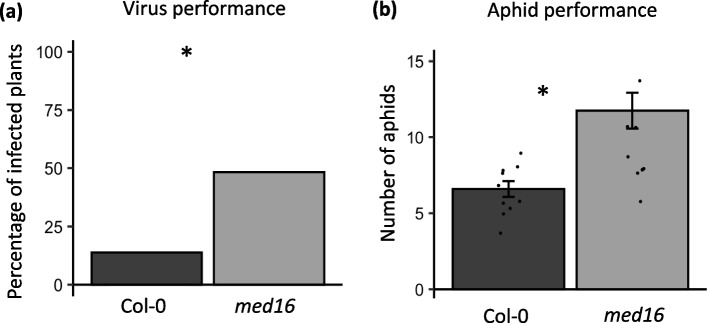
Virus and aphid performance is enhanced on med16 mutant Arabidopsis.(**a**) The percentage of wild type Col-0 and *med16* mutant Arabidopsis plants infected with turnip mosaic virus (TuMV-GFP) 10 days post inoculation. (**b**) The number of progenies produced by a single one-day old *Myzus persica*e foundress was counted on wild type Col-0 plants and *med16* mutants after 9 days. Significant differences were determined for (a) using a Chi-square test. (b) Aphid data was analyzed using a one-way ANOVA and means separation was performed using Tukey HSD test. Asterisk indicates significant differences among treatments using a *p*-value < 0.05, and *N* = 30 for (a) and *N* = 10–12 for (b); error bars indicate standard error

In contrast, the aphid inducible plant defense gene *VSP2,* which is not dependent on MED16, was not induced by virus infection (Fig. 4b; Main effect, virus: F_1,22_ = 3.106, *p*-value = 0.06), and there was no interacting effect of viral infection and aphid feeding (Fig. 5c; virus x aphid: F_1,22_ = 1.677, *p*-value = 0.209). However, *VSP2* expression was significantly induced in response to aphid feeding (Fig. 5c; Main effect, aphid: F_1,22_ = 35.653, *p*-value < 0.0001). *VSP2* expression in plants infested with aphids alone (24.0 ± 4.38) and aphid-infested with virus infection (43.4 ± 8.53) was significantly higher than in uninfested plants (0.718 ± 0.281) or plants with virus infestation only (6.90 ± 1.29) (Fig. 4b).

### Performance of both virus and aphids are higher in the absence of MED16

Next, we evaluated the percentage of plants infected with TuMV after 10 days and the performance of aphids after 9 days of infestation on Col-0 wild type and *med16* mutant Arabidopsis. The results showed that the percentage of *med16* plants infected with TuMV was significantly higher compared to the wild type Col-0 group (Fig. 5a; χ^2^ value = 10.17; *p*-value = 0.001428). Additionally, the performance of aphids was significantly higher on *med16* mutant plants compared to the wild type Col-0 group (Fig. 5b; F_1,20_ = 13.87; *p*-value = 0.00134). These findings suggest that MED16 plays a significant role in the defense mechanisms of Arabidopsis against both TuMV and aphids.

### Aphid feeding in the presence of NIa-Pro increases Med16 abundance and cytosolic cleavage

**Fig. 6:**
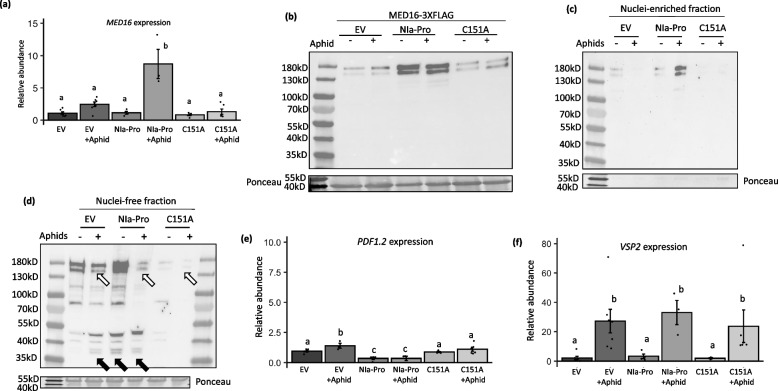
Aphid feeding in presence of NIa-Pro increases Med16 abundance and cleavage outside the nucleus. Relative expression of *MED16* (**a**), PDF1.2 (**e**) and VSP2 (**f**) transcripts in plants expressing the empty expression plasmid (EV), overexpressing NIa-Pro, or the NIa-Pro (C151A) mutant that lacks protease activity, with and without aphid feeding. *UBIQUITIN* was used as an endogenous control, while mock or EV treatments were used as the treatment controls for calculating relative expression. qRT-PCR data were analyzed using two-way ANOVAs and means separation was performed using Tukey test (*N* = 3—7). Bars with different letters indicate significant differences at *p*-value < 0.05; error bars indicate standard error. Protein abundance of *MED16* was measured in *med16* mutants that were complemented with MED16-3XFLAG using immunoblots and $\text{FLAG}$ antibodies in complemented plants were crossed with plants overexpressing NIa-Pro, EV, or NIa-Pro C151A mutant with or without aphid feeding (**b**). Immunoblot detection of the MED16 protein in the nuclei-enriched (**c**) and nucleus-free fractions (**d**) of MED16-3XFLAG complemented Arabidopsis plants. with and without aphid feeding and overexpressing the empty vector (EV), NIa-Pro, or the protease mutant NIa-Pro (C151A) were also measured. The ~ 36kDA cleaved product of MED16 is indicated by black arrows. Each lane represents proteins extracted from 6 individual plants. An equal amount of protein was loaded in each well as shown by Ponceau stain, and MED16 was detected using a FLAG antibody. Each lane represents proteins extracted from 5 individual plants. Equal amounts of protein were loaded into each well as shown by Ponceau stain

We next evaluated if NIa-Pro alone can increase MED16 transcript and protein levels using transgenic NIa-Pro plants that had been crossed with the MED16-FLAG complemented *med16* mutants. In contrast to the TuMV experiments above, aphid infestation increased *MED16* mRNAs (Main effect, aphid: F_2,26_ = 16.92, *p*-value < 0.0001; Fig. 6a). After performing a means separation using Tukey test we observed that Arabidopsis plants expressing NIa-Pro in presence of aphid feeding had significantly higher levels of *MED16* transcripts (8.71 ± 2.26) than all other treatments, including the plants expressing the NIa-Pro protease mutant (C151A) with aphid feeding (1.32 ± 0.409) or without aphid feeding (0.834 ± 0.139) (plant genotype x aphid: F_2,26_ = 17.64, *p*-value < 0.0001; Fig. 6a). While MED16 transcript levels were not elevated in plants expressing NIa-Pro without aphids compared to the controls (Fig. 6a), MED16 protein levels were elevated in Arabidopsis expressing NIa-Pro with or without aphids, and much greater than all other treatments (Fig. 6b). In this experiment, the ~ 36kD protein product was not detected (Fig. 6b). Overall, the highest amount of MED16 was observed in the plants expressing NIa-Pro in presence of aphid feeding, while MED16 levels were the lowest for plants expressing the NIa-Pro C151A mutant with or without aphid feeding (Fig. 6b). Taken together, this shows while NIa-Pro protease activity is required to increased MED16 protein levels, NIa-Pro alone is not sufficient to increase MED16 cleavage. The absence of the cleavage product in plants overexpressing functional NIa-Pro is contrary to what we observe in plants infected with virus. This could be due to faster turnover of the cleaved product in the absence of full viral infection or due to lower levels of the cleaved product detected in the total cell lysate. Therefore, we proceeded to fractionate the cell lysate into nuclear and cytosolic fractions.

As with the virus infection, accumulation of the full-length MED16 in the nuclei-free fraction was the lowest for the NIa-Pro plants with aphid feeding (Fig. 6d; white arrows). Aphid feeding alone also reduced the accumulation of full-length MED16 outside the nucleus, while NIa-Pro expression enhanced it (Fig. 6d), a pattern that was also observed in the nucleus (Fig. 6b). Despite the fact that only NIa-Pro expression had a large impact on the accumulation of full-length MED16, accumulation of the ~ 36 kD putative cleaved product was observed in plants with aphid feeding only and in NIa-Pro expressing plants with or without aphid feeding (Fig. 6d). We did not observe the cleaved MED16 product in the total cell lysate (Fig. 6b). After separation of the nuclei-enriched and nuclei-free cytosolic fractions (Fig. 6c,d) and reconstituting them in the same mass to volume ratio as the total cell lysate before fractionation, we were able to observe the MED16 protein, both intact and the cleaved product, much more clearly for some unknown reason. Full-length MED16 and putative cleavage products were also measured in the nucleus and cytosol of transgenic plants expressing the NIa-Pro C151A mutant with and without aphid feeding (Fig. 6b, d). Levels of full-length MED16 were extremely low in the nucleus in the NIa-Pro C151A plants regardless of aphid presence (Fig. 6d), and similar to levels in aphid control plants (EV + aphids; Fig. 6d). Notably, the ~ 36 kD putative MED16 cleavage product was not detected in either the nucleus or the cytosol for any of the NIa-Pro protease mutant treatments (Fig. 6c, d). In combination, these results suggest that aphids and NIa-Pro induce MED16 nuclear localization and cleavage in the cytosol, potentially reducing the amount of MED16 in the nucleus that can activate defense.

We also measured the transcript abundance of the two marker genes downstream of MED16 in the NIa-Pro over-expressor lines. Notably, aphid feeding had no main effect on the expression of *PDF1.2* in any of these over-expressor lines (Fig. 6e), however there was a significant effect of the plant genotype (Fig. 6e; Main effect, plant genotype: F_1,20_ = 16.811, *p*-value < 0.00001) and an interactive effect of aphids and NIa-Pro on *PDF1.2* expression (Fig. 6e; aphid x plant genotype: F_1,26_ = 4.591, p-value = 0.0446). Post hoc analyses with means separation further show that aphid feeding on empty vector (EV) expressing plants had the highest *PDF1.2* transcript levels (1.39 + / 0.205). In contrast, Arabidopsis plants that overexpress NIa-Pro alone or NIa-Pro with aphids had lower transcript levels for *PDF1.2* (0.341 ± 0.097 and 0.340 ± 0.170 respectively) compared to both mock plants alone (0.937 ± 0.079) and to plants overexpressing NIa-Pro C151A mutant alone or with aphids (0.861 ± 0.042 and 1.10 ± 0.165 respectively; Fig. 6e). Further, *PDF1.2* transcript levels were not significantly different from the EV controls for the plants expressing NIa-Pro C151A (Fig. 6e).

Similarly, in the over-expressor lines, the only significant effect main on *VSP2* expression was from aphid infestation (Fig. 6f; Main effect, aphid: F_1,26_ = 18.59, *p*-value = 0.0002; Main effect, plant genotype: F_1,26_ = 0.038, *p*-value = 0.962) or the interaction of aphid feeding and viral protein expression in plants (Fig. 5d; plant genotype x aphid: F_1,26_ = 0.129, *p*-value = 0.879). The *VSP2* gene expression in aphid-fed plants of either empty vector (EV) or the NIa-Pro over-expressor lines (Fig. 6f; EV = 27.2 ± 8.06; NIa = 33.0 ± 8.23; NIa C151A mutant = 23.6 ± 11.1) were all higher than those that were not infested with aphids (Fig. 6f; EV = 2.06 ± 1.06; NIa = 3.21 ± 1.49; NIa C151A mutant = 1.95 ± 0.251). This suggests that cleavage of MED16 protein by NIa-Pro does not affect components of this MED16-independent plant defense pathway.

## Discussion

The viral effector NIa-Pro was previously shown to enhance aphid performance by suppressing aphid-induced plant defenses downstream of the ethylene signaling pathway [7, 28]. In this study, we demonstrate that a key regulator of ethylene-dependent defenses, MED16, is cleaved in the presence of TuMV (Fig. 2d & 3d), and that NIa-Pro’s protease activity is key to changes in MED16 abundance (Fig. 6b-d), plant defenses (Fig. 4, 6e,f), and enhanced aphid performance (Fig. 1 a,b). NIa-Pro is a cysteine protease that contains a cysteine residue at its active site and has been shown to self-cleave itself from the viral polyprotein and then subsequently cleave other viral proteins from the polyprotein [36, 54]. In virus-infected plants, NIa-Pro is fused with another viral protein, VPg, which contains a NLS and accumulates in the nucleus [55]. In the nucleus, NIa-Pro has also been shown to have DNase activity [56], and it has been predicted that it interacts with plant defense components previously [54, 57, 58].

Viral proteases have been shown to cleave host proteins in several animal systems [23, 24], and more recently, NIa-Pro was shown to be able to cleave plant host proteins [22]. In this study, over 90 proteins in the proteome of different plants (Arabidopsis, *Prunus persica* and *N. benthamiana*) were identified to have NIa-Pro cleavage sites, and cleavage was verified for a few candidates [22]. However, the biological relevance of these proteins remains largely unknown. Our work goes further by demonstrating viral and aphid performance was enhanced on *med16* mutants (Fig. 5b), and that the NIa-Pro protease activity was required for defense suppression (Fig. 6e, f). These findings provide insights into the mechanisms underlying virus-aphid-plant interactions and have implications for plant defense strategies against viral infections and insect herbivory.

In a previous study, we demonstrated that NIa-Pro re-localizes from the nucleus to the vacuole in the presence of aphid feeding. When an NLS was added to NIa-Pro, it was confined completely to the nucleus and was unable to re-localize, suppress plant defenses, or increase aphid performance on host plants [29]. This suggested that NIa-Pro's role in plant-aphid interactions occurs outside of the nucleus, consistent with the current study's findings. The nucleus contains pores that allow the entry and exit of a 50 kDa protein such as NIa-Pro [29]. MED16, which is needed for transcription regulation, is ~ 140kD and is too large to enter the nucleus without a NLS [59]. In our MED16 immunoblot assays, we observed two closely placed bands of the intact MED16 protein in control plants, as did Wang et al. in their study [30]. This could be either due to the presence of isoforms of MED16 (Fig S4b) or due to cleavage by another plant protease that is not known. Regardless, in the previous studies there was no evidence of a putative cleaved MED16 product of ~ 36kD as we observed in virus-infected and NIa-Pro overexpressing plants (Fig. 2d, 3d). We did find a potential NLS at the C-terminus of MED16, immediately after one of the putative NIa-Pro cleavage sites (Fig. 2d; 3c, d). This suggests NIa-Pro inhibits MED16’s function by removing the NLS in the cytosol and perhaps preventing nuclear localization. This is further supported by the detection of the predicted ~ 36 KD putative cleavage product only in the cytosol fractions from TuMV-infected plants and NIa-Pro expressing plants (Fig. 3 c, d). Furthermore, MED16 was only present in the nucleus as a full-length protein (Fig. 3 a, b). We also see the same result in *Nicotiana benthamiana*, where MED16 protein was cleaved in the presence of virus infection at a VLPE site, leading to the removal of the NLS signal leading to the generation of a 21kD fragment of MED16 (Fig S5). In Arabidopsis, in the presence of virus infection and aphid feeding, there is a significant level of the putative cleaved MED16 protein detected in the cytosol. In contrast, while very low amounts of intact protein were found in the nucleus, suggesting that reduced levels of intact MED16 in the nucleus prevented downstream defenses against aphids and viruses to be activated as seen in PDF1.2 transcript abundance (Fig. 4a). In plants overexpressing NIa-pro, we observed higher levels of intact MED16 in the nuclei-enriched fraction with aphid feeding; however, the level of full-length MED16 protein expression in the nucleus was similar in NIa-pro overexpressor and control plants without aphid feeding (Fig. 6c). Notably, there was also more cleaved MED16 product in the nuclei-free cytosolic fraction of plants overexpressing NIa-Pro both with or without aphid feeding as well as in plants with just aphid feeding (Fig. 6d). Although the putative cleaved MED16 fragment of ~ 36kD has the NLS signal, it did not relocate to the nucleus (Fig. 3 c,d;6 -b-d). We are not sure why the cleaved product does not relocate to the nucleus, but these results suggest that either the relocation of MED16 to nucleus can occur only in full-length form or that the protein was below the detectable limit of immunoblot assays. Another possible explanation could be that NIa-pro interacts with other plant proteins that are involved in organellar transport. Importin a and Importin b are plant proteins present in the cytosol that have been shown to bind NLS signals of several regulatory plant proteins and shuttle them into the nucleus [60]. The interaction between MED16, NIa-Pro, and such plant organellar shuttle protein Importin remains to be studied.

To our knowledge, this study is the first example of a plant virus protease, and any plant pathogen effector, that induces a plant regulatory protein abundance and enhances the removal of the plant protein’s NLS signal to increase virulence. In animal viruses, however, it has been shown that viral proteases can remove NLS from animal proteins. For example, the viral protease 3C^pro^ of Foot-and-mouth disease virus (FMDV) can cleave the C-terminus end of host RNA-binding protein Sam68 that houses the NLS signal. The cleavage of Sam68 prevents it from being localized in the nucleus and its subsequent localization in the cytoplasm, thereby increasing the viral titer 1000-fold in fetal porcine cell lines [43]. The sugar beet cyst nematode *Heterodera schachttii* secretes the 4E02 effector in host plants, which then interacts with the vacuolar plant protease RD21A (RESPONSIVE TO DEHYDRATION 21A). RD21A is a cysteine protease that moves to the nucleus when bound with 4E02 and is predicted to cleave several plant defense-related proteins rendering the plants susceptible to *H. schachttii* as well as to necrotrophic pathogen *Botrytis cinerea* [61]. This work shows how a microbial effector can highjack a host protease to relocate it to the nucleus to cleave defense-related plant proteins. However, no other phytopathogen effectors have been shown to target a host regulatory protein by enhancing the removal of the NLS from the host protein to disrupt their functions.

**Fig. 7:**
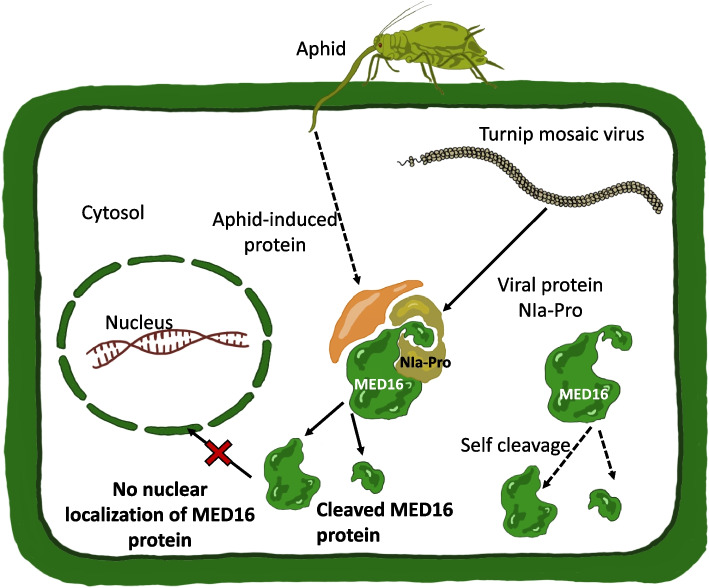
Model of MED16 cleavage in virus infected and aphid infested plants. A working model of how MED16 protein is plants is cleaved in presence of aphid and virus. The viral effector NIa-Pro cleaves MED16 protein in presence of an aphid-induced plant protein. The protease activity of NIa-Pro is essential to suppress aphid and pathogen-related defense gene PDF1.2, that is downstream of MED16. Nuclear localization of MED16 by its cleavage occurs in presence of both NIa-Pro and aphids, therefore, an unknown aphid-induced protein may be facilitating cleavage of NIa-Pro and thereby suppressing virus and aphid related plant defenses downstream of MED16

While the greatest amount of MED16 cleavage product was detected in the presence of virus and aphids (Fig. 3d), and in the presence of NIa-Pro and aphids in the cytosol (Fig. 6d), the cleavage product was also detected in transgenic control plants infested with aphids alone in the cytosol (Fig. 6d). Similarly, the MED16 cleavage product was detected in low amounts in the mock-inoculated plants with aphids using total protein extracts (Fig. 2d). This suggests that either some unknown aphid effector or an aphid-induced plant protease may facilitate the cleavage of MED16. This leads us to believe that the protease activity of NIa-Pro may not be sufficient to cleave MED16 and that it may need the help of another aphid-induced protease to cleave MED16 (Fig. 7). Subsequent studies need to be done to identify the presence of such protease. We also observe some amount of intact MED16 in the nucleus in the presence of virus and aphid or in the presence of NIa-Pro and aphid (Fig. 3 b-d). However, apparently, the presence of this small amount of MED16 in the nucleus was not enough to induce the downstream defense gene PDF1.2 (Fig. 4a; 6e).

Plants, insects, and microbes have been co-evolving for millions of years. During this process, they have developed the ability to recognize each other as either beneficial partners or antagonists. As plants cannot move, they rely heavily on the recognition of chemical signals from microbes and insects to differentiate between beneficial and harmful interactions and respond accordingly. One type of signal plants have evolved to recognize is the peptides created when pathogen and herbivore proteins are broken down by the host plant or by the colonizing organisms themselves [62, 63]. It was shown that cells of tomato and Arabidopsis plants can perceive nanomolar concentrations of only a 15 amino acid fragment of the bacterial flagellin protein [64]. Plants can also recognize Inceptin, a 11 amino acid cleaved peptide fragment of chloroplast ATPase, that was shown to induce defenses against caterpillars in cowpea [65]. It is not known if MED16 cleavage products are recognized by host plants and used as a signal to upregulate defenses in adapted hosts. However, this work paves the way for future work on signal recognition and defense response in plant-virus-interactions, as well as on regulation of the MED16 protein.

## Supplementary Information

Additional file 1. Figure S1. RT-PCR gel showing presence of NIa-Pro transcript in (a) Arabidopsis Col-0 plants that were rub-inoculated with TuMV-GFP, (b) Arabidopsis plants that were overexpressing the empty plasmid vector (EV), NIa-Pro or NIa-Pro C151 mutant, or (c) Arabidopsis mutants complemented with MEG16-3XFLAG and crossed with plants overexpressing the empty plasmid vector (EV), NIa-Pro, or NIa-Pro C151 mutant for the T2 generation (pool of N=6). The amplified product from C151A was sequenced to verify the C151A mutation in the NIa-Pro mutant over-expressor. The primers used amplify a 299 bp amplicon of NIa-Pro from TuMV at the 5’-end of the gene. Figure S2. Proteins extracted from the nuclei-free and nuclei-enriched fractions of Arabidopsis plants that were (a, b) mock or virus-infected or (c, d) expressing the empty expression vector (EV), NIa-Pro, or the protease mutant NIa-Pro C151A, and expressing MED16:3XFLAG. Pure organellar separations were verified by immunodetection of (a, c) the nucleus specific ~17kD histone H3 protein histone H3 antibodies and (b, d) the cystosol specific ~105kD phosphoenol pyruvate carboxylase or PEPC (b, d). Figure S3. Sequence comparison of the three predicted isoforms of MED16 (AT4G04920). Figure S4. AT4G04920.3 is the most abundant isoform of MED16.(a) Isoform abundance of MED16 AT4G04920.1, AT4G04920.2, and AT4G04920.3 in Arabidopsis plants with and without TuMV infection and aphid feeding. (b) The predicted protein structures of the different MED16 isoforms (AT4G04920.1, AT4G04920.2, and AT4G04920.3) using I-TASSER. Figure S5. The *Nicotiana benthamiana* MEDIATOR16 (MED16) protein is cleaved upstream of a nuclear localization signal (NLS) in the presence of turnip mosaic virus. (a) The *Nicotiana benthamiana* MED16 protein sequence was searched for the two most common NIa-Pro cleavage sites (VxxQ & VxxE). Predicted cleavage product sizes are shown. (b) The location of the NIa-Pro cleavage sites (Green and Blue) and of the nuclear localization signal (NLS; Yellow) in the amino acid sequence are presented. (c) *N.benthaminana* plants were co-infiltrated with *Agrobacterium tumefaciens *expressing the virulent turnip mosaic virus with GFP and the pMDC32 vector expressing MED16 gene from *Nicotiana benthamiana* MYC tag in the N-terminal end and 6XHIS tag at the C-terminal end (MYC-NbMED16-HIS). The protein abundance of *MED16* was measured using immunoblots and MYC and HIS antibodies. The full length MED16 protein (white arrows) was detected as well as a smaller ~22kDa cleaved product (black arrows). Each lane represents proteins extracted from 5 individual plants. Equal amounts of protein were loaded into each well as shown by Ponceau stain. Figure S6. Standard curve of qRT-PCR *UBIQUITIN* primer used to quantify relative abundance of *PDF1.2, VSP2* and *MED16* transcripts.

Additional file 2. Supplemental Table S1. List or primers used. Supplemental Table S2. Band intensities of MED16 protein of immunoblots in Figures 1-3

## Data Availability

The datasets used and analyzed in the current study are available from the corresponding author upon request.

## Abbreviations

TuMV: Turnip Mosaic Virus
NIa-Pro: Nuclear inclusion protease a
MED 16: Mediator 16 protein
NLS: Nuclear localization signal
HIS: Histidine tag
MYC: Myc antigenic epitope tag
FLAG: Flag antigenic epitope tag
EV: Empty vector
GFP: Green fluorescent protein
PDF1.2: Plant Defensin 1.2 gene
VSP2: Vegetative storage protein 2 gene
