# Supplementary material for: Turnip mosaic virus infection cleaves MEDIATOR SUBUNIT16 in plants increasing plant susceptibility to the virus and its aphid vector Myzus persicae
Source: BMC Plant Biol. 2025 Apr 2;25:411. doi: 10.1186/s12870-025-06411-2 (PMC11963320; doi:10.1186/s12870-025-06411-2)
Supplement: Supplementary file 1 — Additional file 1. Figure S1. RT-PCR gel showing presence of NIa-Pro transcript in (a) Arabidopsis Col-0 plants that were rub-inoculated with TuMV-GFP, (b) Arabidopsis plants that were overexpressing the empty plasmid vector (EV), NIa-Pro or NIa-Pro C151 mutant, or (c) Arabidopsis mutants complemented with MEG16-3XFLAG and crossed with plants overexpressing the empty plasmid vector (EV), NIa-Pro, or NIa-Pro C151 mutant for the T2 generation (pool of N=6). The amplified product from C151A was sequenced to verify the C151A mutation in the NIa-Pro mutant over-expressor. The primers used amplify a 299 bp amplicon of NIa-Pro from TuMV at the 5’-end of the gene. Figure S2. Proteins extracted from the nuclei-free and nuclei-enriched fractions of Arabidopsis plants that were (a, b) mock or virus-infected or (c, d) expressing the empty expression vector (EV), NIa-Pro, or the protease mutant NIa-Pro C151A, and expressing MED16:3XFLAG. Pure organellar separations were verified by immunodetection of (a, c) the nucleus specific ~17kD histone H3 protein histone H3 antibodies and (b, d) the cystosol specific ~105kD phosphoenol pyruvate carboxylase or PEPC (b, d). Figure S3. Sequence comparison of the three predicted isoforms of MED16 (AT4G04920). Figure S4. AT4G04920.3 is the most abundant isoform of MED16.(a) Isoform abundance of MED16 AT4G04920.1, AT4G04920.2, and AT4G04920.3 in Arabidopsis plants with and without TuMV infection and aphid feeding. (b) The predicted protein structures of the different MED16 isoforms (AT4G04920.1, AT4G04920.2, and AT4G04920.3) using I-TASSER. Figure S5. The Nicotiana benthamiana MEDIATOR16 (MED16) protein is cleaved upstream of a nuclear localization signal (NLS) in the presence of turnip mosaic virus. (a) The Nicotiana benthamiana MED16 protein sequence was searched for the two most common NIa-Pro cleavage sites (VxxQ & VxxE). Predicted cleavage product sizes are shown. (b) The location of the NIa-Pro cleavage sites (Green and Blue) and of the nu [file 12870_2025_6411_MOESM1_ESM.pptx]

## Slide 1
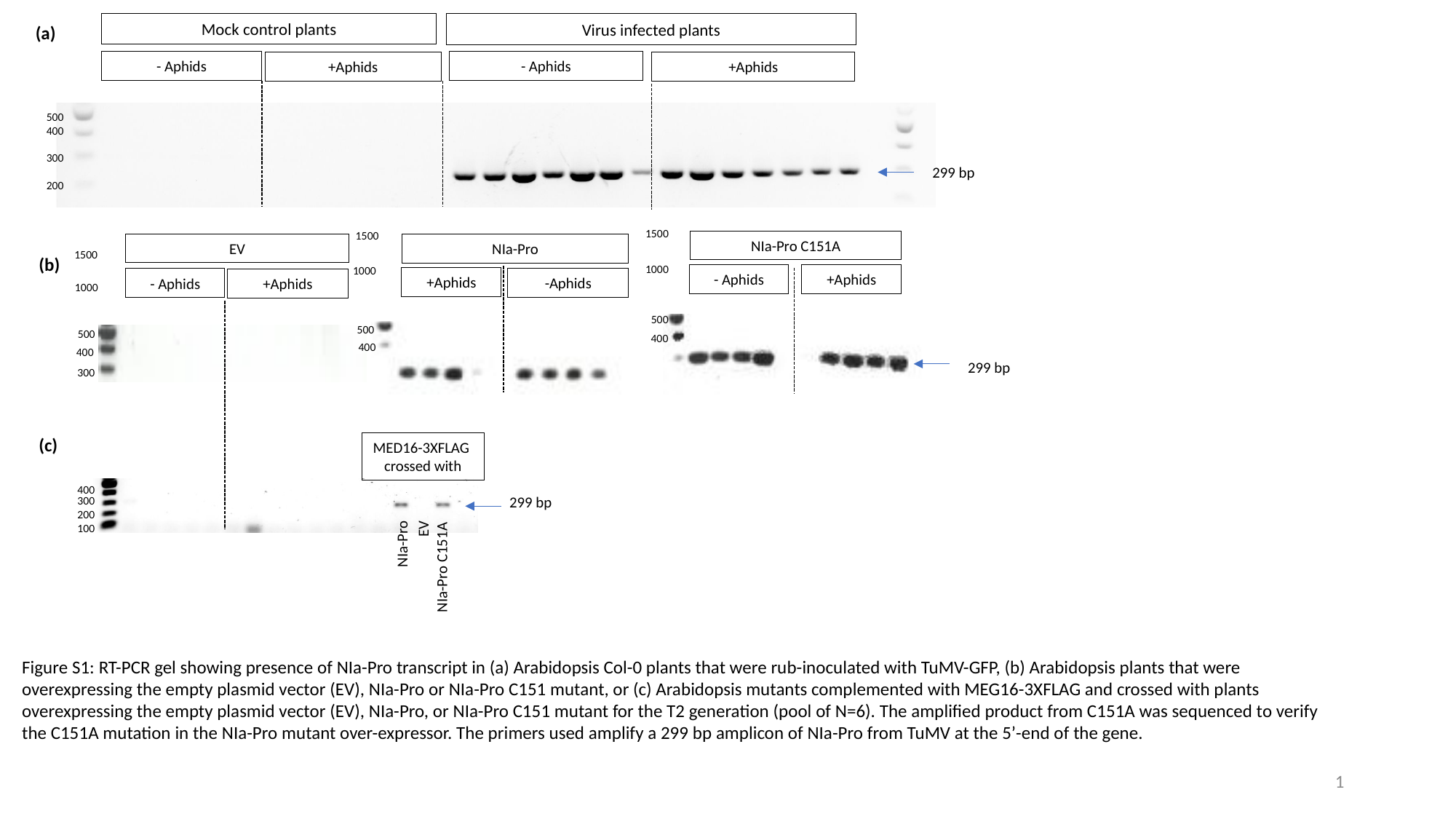

Mock control plants
Virus infected plants
- Aphids
- Aphids
+Aphids
+Aphids
500
400
300
200
299 bp
(a)
1500
NIa-Pro C151A
1000
+Aphids
- Aphids
500
400
1500
NIa-Pro
1000
+Aphids
-Aphids
500
400
EV
1500
- Aphids
+Aphids
500
400
300
(b)
1000
299 bp
(c)
MED16-3XFLAG
crossed with
300
200
100
EV
NIa-Pro
NIa-Pro C151A
400
299 bp
Figure S1: RT-PCR gel showing presence of NIa-Pro transcript in (a) Arabidopsis Col-0 plants that were rub-inoculated with TuMV-GFP, (b) Arabidopsis plants that were overexpressing the empty plasmid vector (EV), NIa-Pro or NIa-Pro C151 mutant, or (c) Arabidopsis mutants complemented with MEG16-3XFLAG and crossed with plants overexpressing the empty plasmid vector (EV), NIa-Pro, or NIa-Pro C151 mutant for the T2 generation (pool of N=6). The amplified product from C151A was sequenced to verify the C151A mutation in the NIa-Pro mutant over-expressor. The primers used amplify a 299 bp amplicon of NIa-Pro from TuMV at the 5’-end of the gene.
1

## Slide 2
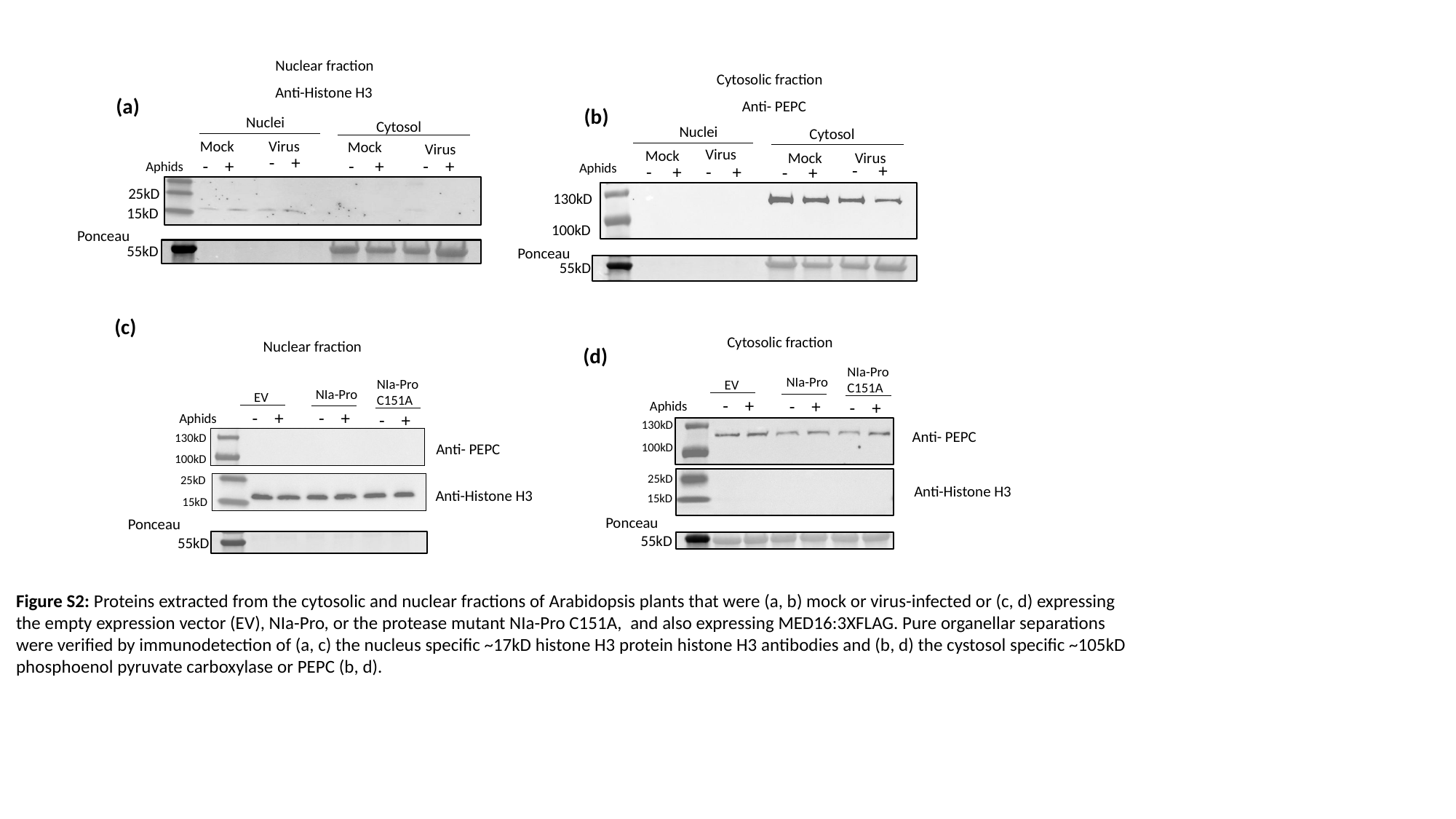

Nuclear fraction
Cytosolic fraction
Anti- PEPC
(b)
Nuclei
Cytosol
Virus
Mock
Virus
Mock
- +
Aphids
- +
- +
- +
130kD
100kD
Ponceau
(d)
NIa-Pro
C151A
NIa-Pro
EV
- +
- +
- +
Aphids
130kD
100kD
25kD
15kD
Ponceau
55kD
55kD
Cytosolic fraction
Anti- PEPC
Anti-Histone H3
Anti-Histone H3
(a)
Nuclei
Cytosol
Virus
Mock
Mock
Virus
- +
- +
- +
- +
Aphids
25kD
15kD
Ponceau
(c)
NIa-Pro
C151A
NIa-Pro
EV
- +
- +
- +
Aphids
130kD
100kD
25kD
15kD
Ponceau
55kD
Nuclear fraction
Anti- PEPC
Anti-Histone H3
55kD
Figure S2: Proteins extracted from the cytosolic and nuclear fractions of Arabidopsis plants that were (a, b) mock or virus-infected or (c, d) expressing the empty expression vector (EV), NIa-Pro, or the protease mutant NIa-Pro C151A, and also expressing MED16:3XFLAG. Pure organellar separations were verified by immunodetection of (a, c) the nucleus specific ~17kD histone H3 protein histone H3 antibodies and (b, d) the cystosol specific ~105kD phosphoenol pyruvate carboxylase or PEPC (b, d).

## Slide 3
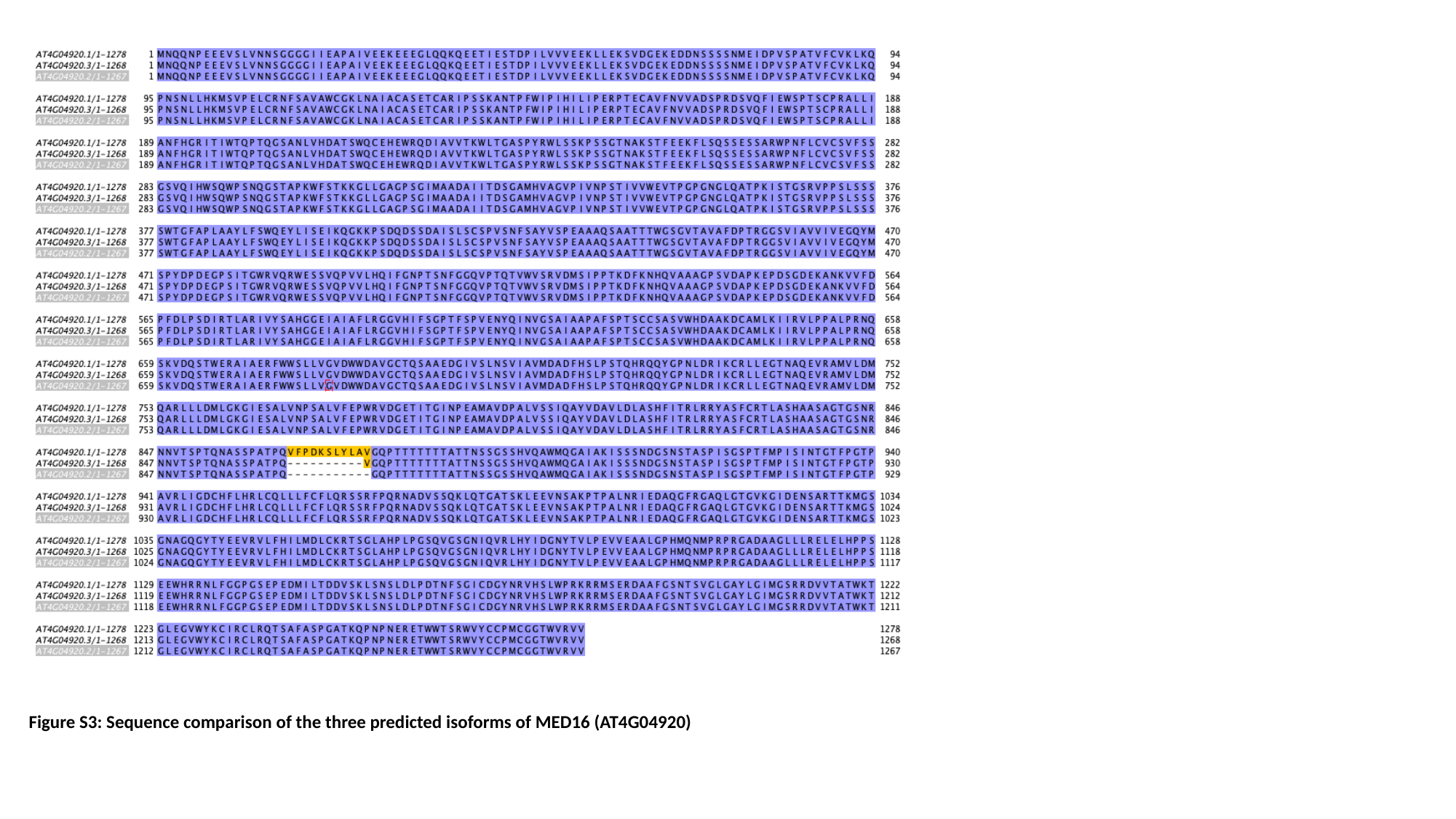

Figure S3: Sequence comparison of the three predicted isoforms of MED16 (AT4G04920)

## Slide 4
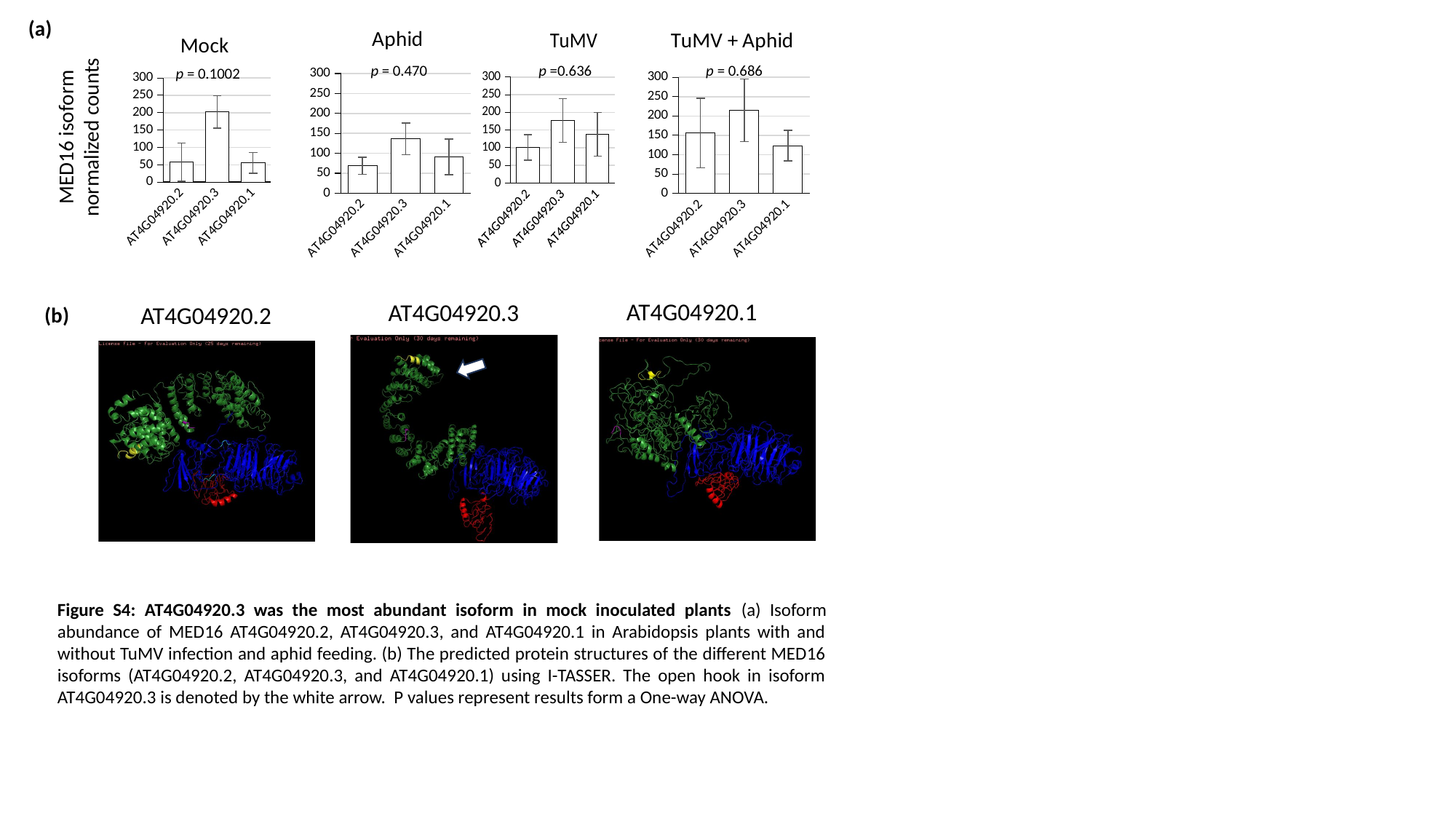

(a)
### Chart: Aphid
| Category | |
|---|---|
| AT4G04920.2 | 68.97519999999999 |
| AT4G04920.3 | 135.92243333333334 |
| AT4G04920.1 | 91.43568333333333 |
### Chart: TuMV
| Category | |
|---|---|
| AT4G04920.2 | 100.88376666666666 |
| AT4G04920.3 | 176.97410000000002 |
| AT4G04920.1 | 138.47580000000002 |
### Chart: Mock
| Category | |
|---|---|
| AT4G04920.2 | 57.392261062193334 |
| AT4G04920.3 | 202.15099999999998 |
| AT4G04920.1 | 55.12350333333334 |
### Chart: TuMV + Aphid
| Category | |
|---|---|
| AT4G04920.2 | 156.1076666666667 |
| AT4G04920.3 | 214.8584 |
| AT4G04920.1 | 123.03383333333333 |p = 0.686
p = 0.470
p =0.636
p = 0.1002
MED16 isoform
normalized counts
AT4G04920.1
AT4G04920.3
AT4G04920.2
(b)
P = 0.1002
Figure S4: AT4G04920.3 was the most abundant isoform in mock inoculated plants (a) Isoform abundance of MED16 AT4G04920.2, AT4G04920.3, and AT4G04920.1 in Arabidopsis plants with and without TuMV infection and aphid feeding. (b) The predicted protein structures of the different MED16 isoforms (AT4G04920.2, AT4G04920.3, and AT4G04920.1) using I-TASSER. The open hook in isoform AT4G04920.3 is denoted by the white arrow. P values represent results form a One-way ANOVA.

## Slide 5
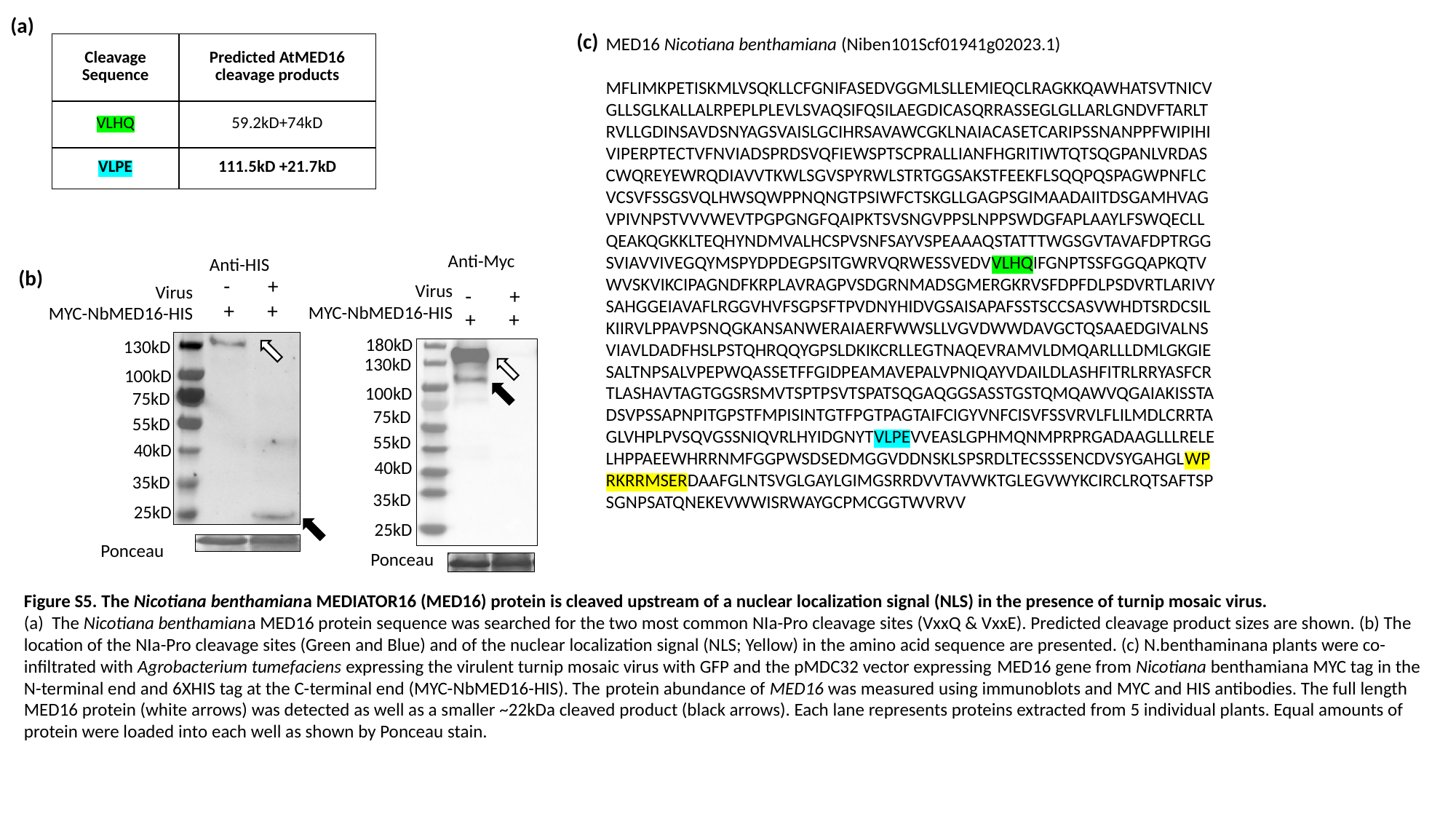

(a)
(c)
MED16 Nicotiana benthamiana (Niben101Scf01941g02023.1)
MFLIMKPETISKMLVSQKLLCFGNIFASEDVGGMLSLLEMIEQCLRAGKKQAWHATSVTNICVGLLSGLKALLALRPEPLPLEVLSVAQSIFQSILAEGDICASQRRASSEGLGLLARLGNDVFTARLTRVLLGDINSAVDSNYAGSVAISLGCIHRSAVAWCGKLNAIACASETCARIPSSNANPPFWIPIHIVIPERPTECTVFNVIADSPRDSVQFIEWSPTSCPRALLIANFHGRITIWTQTSQGPANLVRDASCWQREYEWRQDIAVVTKWLSGVSPYRWLSTRTGGSAKSTFEEKFLSQQPQSPAGWPNFLCVCSVFSSGSVQLHWSQWPPNQNGTPSIWFCTSKGLLGAGPSGIMAADAIITDSGAMHVAGVPIVNPSTVVVWEVTPGPGNGFQAIPKTSVSNGVPPSLNPPSWDGFAPLAAYLFSWQECLLQEAKQGKKLTEQHYNDMVALHCSPVSNFSAYVSPEAAAQSTATTTWGSGVTAVAFDPTRGGSVIAVVIVEGQYMSPYDPDEGPSITGWRVQRWESSVEDVVLHQIFGNPTSSFGGQAPKQTVWVSKVIKCIPAGNDFKRPLAVRAGPVSDGRNMADSGMERGKRVSFDPFDLPSDVRTLARIVYSAHGGEIAVAFLRGGVHVFSGPSFTPVDNYHIDVGSAISAPAFSSTSCCSASVWHDTSRDCSILKIIRVLPPAVPSNQGKANSANWERAIAERFWWSLLVGVDWWDAVGCTQSAAEDGIVALNSVIAVLDADFHSLPSTQHRQQYGPSLDKIKCRLLEGTNAQEVRAMVLDMQARLLLDMLGKGIESALTNPSALVPEPWQASSETFFGIDPEAMAVEPALVPNIQAYVDAILDLASHFITRLRRYASFCRTLASHAVTAGTGGSRSMVTSPTPSVTSPATSQGAQGGSASSTGSTQMQAWVQGAIAKISSTADSVPSSAPNPITGPSTFMPISINTGTFPGTPAGTAIFCIGYVNFCISVFSSVRVLFLILMDLCRRTAGLVHPLPVSQVGSSNIQVRLHYIDGNYTVLPEVVEASLGPHMQNMPRPRGADAAGLLLRELELHPPAEEWHRRNMFGGPWSDSEDMGGVDDNSKLSPSRDLTECSSSENCDVSYGAHGLWPRKRRMSERDAAFGLNTSVGLGAYLGIMGSRRDVVTAVWKTGLEGVWYKCIRCLRQTSAFTSPSGNPSATQNEKEVWWISRWAYGCPMCGGTWVRVV
| Cleavage Sequence | Predicted AtMED16 cleavage products |
| --- | --- |
| VLHQ | 59.2kD+74kD |
| VLPE | 111.5kD +21.7kD |
Anti-Myc
Anti-HIS
(b)
 - +
 + +
 - +
Virus
MYC-NbMED16-HIS
Virus
MYC-NbMED16-HIS
 + +
180kD
130kD
130kD
100kD
100kD
75kD
75kD
55kD
55kD
40kD
40kD
35kD
35kD
25kD
25kD
Ponceau
Ponceau
Figure S5. The Nicotiana benthamiana MEDIATOR16 (MED16) protein is cleaved upstream of a nuclear localization signal (NLS) in the presence of turnip mosaic virus.
(a) The Nicotiana benthamiana MED16 protein sequence was searched for the two most common NIa-Pro cleavage sites (VxxQ & VxxE). Predicted cleavage product sizes are shown. (b) The location of the NIa-Pro cleavage sites (Green and Blue) and of the nuclear localization signal (NLS; Yellow) in the amino acid sequence are presented. (c) N.benthaminana plants were co-infiltrated with Agrobacterium tumefaciens expressing the virulent turnip mosaic virus with GFP and the pMDC32 vector expressing MED16 gene from Nicotiana benthamiana MYC tag in the N-terminal end and 6XHIS tag at the C-terminal end (MYC-NbMED16-HIS). The protein abundance of MED16 was measured using immunoblots and MYC and HIS antibodies. The full length MED16 protein (white arrows) was detected as well as a smaller ~22kDa cleaved product (black arrows). Each lane represents proteins extracted from 5 individual plants. Equal amounts of protein were loaded into each well as shown by Ponceau stain.

## Slide 6
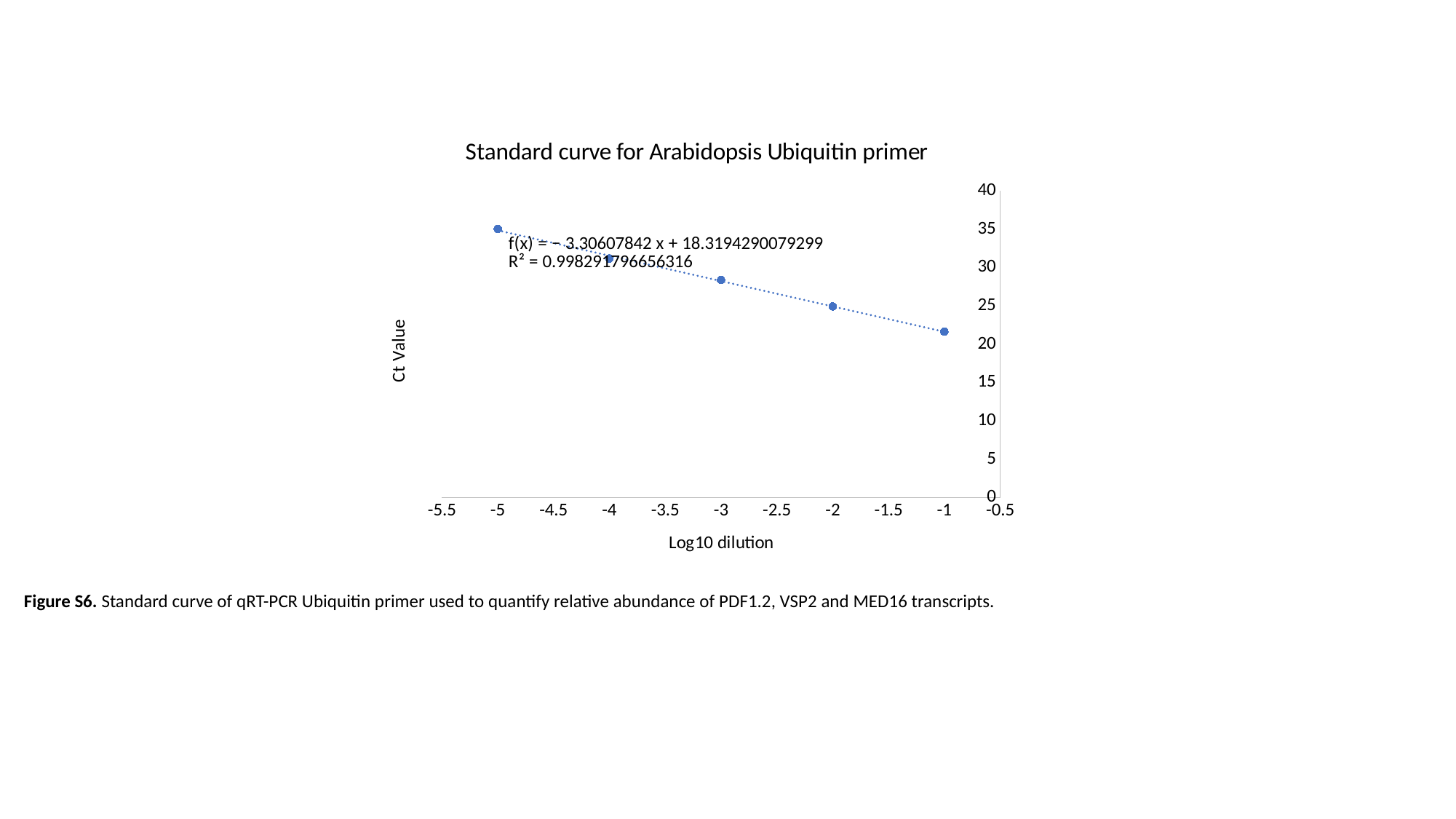

### Chart: Standard curve for Arabidopsis Ubiquitin primer
| Category | CT |
|---|---|Figure S6. Standard curve of qRT-PCR Ubiquitin primer used to quantify relative abundance of PDF1.2, VSP2 and MED16 transcripts.
