# Supplementary material for: Turnip mosaic virus infection cleaves MEDIATOR SUBUNIT16 in plants increasing plant susceptibility to the virus and its aphid vector Myzus persicae
Source: BMC Plant Biol. 2025 Apr 2;25:411. doi: 10.1186/s12870-025-06411-2 (PMC11963320; doi:10.1186/s12870-025-06411-2)
Supplement: Supplementary file 2 — Additional file 2. Supplemental Table S1. List or primers used. Supplemental Table S2. Band intensities of MED16 protein of immunoblots in Figures 1-3 [file 12870_2025_6411_MOESM2_ESM.pdf]

Table S1: List or primers used:

Gene name	Forward Primer	Reverse Primer	Gene ID
Arabidopsis Ubiquitin	AAAGAGATAACAGGAACGGAAACATA	GGCCTTGTATAATCCCTGATGAATAA	AT4G05320
Arabidopsis PDF1.2	TCATTCCGATAGTCGACCAAG	TTGATTTGCGAAATACCGAAC	AT5G44420
Arabidopsis VSP2	CTCATATTGAAGCCTAATGGTTCG	GGGGACAATGCGATGAAGAT	AT5G24770
Arabidopsis MED16	TCTGATCATGAAGCCGGAGAC	CACCAACATCCTCGGAAGCA	AT4G04920
Turnip mosaic virus NIa-Pro	CTCACAAATGTTTCAGATGGAGCAT	CTCTTGGTTTGGAAATTGGACCC	

Supplemental Table S2: Band intensities of MED16 protein of immunoblots in Figures 1-3

Fig 1d	Band Intensity	
	intact NIa	cleaved
Control	61.52	0
Virus	37.37	37.96
Fig 2b		
	intact	cleaved
Mock	20.1	0.68
Mock+Aphid	19.43	0.71
Virus	44.04	3.45
Virus+aphid	55.8	5.03
Fig 2c (nuclear)		
	intact	cleaved
Mock	0.13	0.22
Mock+Aphid	1.31	0.12
Virus	8.49	0.02
Virus+aphid	17.93	0.01
2d (cytosolic)		
	intact	cleaved
Mock	8.75	0.09
Mock+Aphid	8.93	1.09
Virus	3.76	1.43
Virus+aphid	2.26	27.5
Fig 3b		
	intact	Cleaved
EV	5.26	0.02
EV+aphid	13.22	0.01
NIa	67.69	0.08
NIa+Aphid	67.42	0.05
NIa C151A mutant	14.66	0.01
NIa C151A mutant+Aphid	25.51	0.03
Fig 3c(nucleus-enriched)		
EV	13.29	0.01
EV+aphid	1.15	0.02
NIa	11.67	0.03
NIa+Aphid	62.5	0
NIa C151A mutant	3.12	0.01
NIa C151A mutant+Aphid	2.45	0.04
Fig 3d (nucleus-free)		
EV	54.35	0.25
EV+aphid	38.86	4.94
NIa	77.95	12.12
NIa+Aphid	23.85	7.08
NIa C151A mutant	7.49	0.17
NIa C151A mutant+Aphid	4.52	0.02
